# Constructing prediction models and analyzing factors in suicidal ideation using machine learning, focusing on the older population

**DOI:** 10.1371/journal.pone.0305777

**Published:** 2024-07-22

**Authors:** Hyun Woo Jung, Jin Su Jang

**Affiliations:** 1 Department of Health Administration, Graduate School, Yonsei University, Wonju, Republic of Korea; 2 Yonsei Institute of Health and Welfare, Yonsei University Mirae Campus, Wonju, Republic of Korea; 3 Human Behavior & Genetic Institute, Associate Research Center, Korea University, Seoul, Republic of Korea; German University in Cairo, EGYPT

## Abstract

Suicide among the older population is a significant public health concern in South Korea. As the older individuals have long considered suicide before committing suicide trials, it is important to analyze the suicidal ideation that precedes the suicide attempt for intervention. In this study, six machine learning algorithms were employed to construct a predictive model for suicidal thinking and identify key variables. A traditional logistic regression analysis was supplementarily conducted to test the robustness of the results of machine learning. All analyses were conducted using a hierarchical approach to compare the model fit of each model in both machine learning and logistic regression. Three models were established for analysis. In Model 1, socioeconomic, residential, and health behavioral factors were incorporated. Model 2 expanded upon Model 1 by integrating physical health status, and Model 3 further incorporated mental health conditions. The results indicated that the gradient boosting algorithm outperformed the other machine learning techniques. Furthermore, the household income quintile was the most important feature in Model 1, followed by subjective health status, oral health, and exercise ability in Model 2, and anxiety and depression in Model 3. These results correspond to those of the hierarchical logistic regression. Notably, economic and residential vulnerabilities are significant factors in the mental health of the older population with higher instances of suicidal thoughts. This hierarchical approach could reveal the potential target population for suicide interventions.

## Introduction

Suicide is a serious global public health issue [1, 2]. South Korea, in particular, has shown the highest suicide rate among the Organization for Economic Co-operation and Development (OECD) countries for a decade [3, 4]. The suicide rate in South Korea in 2020 was 24.1, which was 2.23 times higher than the OECD standardized population of 11.0 per 100,000 [3, 4]. Meanwhile, it is globally the number of suicide deaths is highest among middle-aged adults in high-income countries [2]. South Korea also shows a similar suicide distribution. The highest number of suicides occurred among individuals in their 50s, and suicide rates per 100,000 people substantially increased from the age of 60 years (28.4%), reaching a peak of 62.6% in the 80s [5].

Suicide in the elderly population exhibits distinct characteristics compared to younger individuals [6]. Younger generations often engage in impulsive suicide attempts, which result in lower completion rate [7]. Contrarily, elderly individuals tend to carefully consider various circumstances and plan their suicide in detail [8], leading to higher completion rate [9–11]. Hence, suicidal ideation has emerged as a potent predictor of suicide attempts, particularly among the elderly. Considering their mutable and time-consuming nature, targeting these ideations could be a pivotal focus of effective suicide prevention interventions.

Previous literature has concentrated on incidents of suicide attempts and completed suicides, and comparatively less on suicidal ideation [6, 12]. Even within the constraints of existing literature, the prevailing view in most studies is to treat suicidal ideation merely as a proxy for suicide attempts or completed suicide. However, recent studies have recognized the importance of suicidal thoughts as a crucial prevention indicator because of their significant role as predictors immediately preceding suicidal attempt [13]. We argue that the significance of suicidal ideation transcends mere prediction; rather, it resides in its meaning. Many public health politicians regard suicide as a mental disease [14]. Nevertheless, the distinguishing characteristic of suicide from conventional diseases lies in the incorporation of individuals’ perspectives and assessments of their life and the social environment in which they are engaged. Therefore, it is necessary to interpret suicidal thoughts in the context of their lives and social environment rather than simply viewing them from the perspective of a mental illness that can be treated with hormonal control and medication.

A majority of related studies have relied on conventional statistical methodologies, including chi-square, multiple logistic regression, multi-level analysis, and similar approaches [6, 9, 12, 13, 15–17]. Furthermore, the variables employed in each research model are significantly different from one another, reflecting the diverse range of related articles distributed across various research domains, such as gerontology, nursing, social behavior, and public health [15–17]. A comprehensive analysis, irrespective of the research domain, requires extensive data that encompasses diverse variables and ensures an ample sample size with representatives for the entire population. In addition, appropriate techniques are required to handle the large prepared datasets. A limitation of traditional statistical techniques is that the number of input variables is limited when conducting regression analysis. To overcome these problems, social science researchers have recently attempted machine learning for various subjects [18–21].

Machine learning algorithms are defined as a process of building computer systems that automatically learn from data, model fitting, and conduct trials in subset data using the fitted model [22]. This algorithm learns data without limiting the number of covariates and suggests the best model. Evidence has been presented indicating that the machine model’s explanatory power surpasses that of traditional statistical techniques, such as multiple regression analysis. It possesses the advantage of deriving ‘feature importance’ through sub-analysis, allowing the determination of variables that are most crucial in predicting the outcome variable. However, a shortcoming of machine learning is its inability to articulate the vectors of dependent variables according to the specific levels of the independent variables. Specifically, machine learning can identify marital status as the most important variable for predicting suicidal ideation. However, it does not distinguish whether married, unmarried, or divorced individuals are more likely to commit suicide. Therefore, this study enhances the robustness of machine learning modeling results by supplementing them with a chi-square test and multiple logistic regression analysis.

It is commonly accepted that suicide is determined not only by psychological factors [16], but also by socioeconomic status, physical health and health behavior [12]. Furthermore, recent studies have emphasized on the macro-level factors surrounding each individual [23, 24]. Han and Lee [12] employed multilevel analyses, including individual, household, and administrative area variables. However, machine learning cannot distinguish the macro level from individual-set data. Therefore, we decided to include individuals and households at the mezzo level. Meanwhile, existing studies did not include sufficient residential factors, such as the type of house, home ownership, and residential location. Residential factors provide a rich interpretation of environmental factors, economic symbols, and social status.

The purpose of this study was to derive the most appropriate model and important features of suicidal ideation for the older population by using machine learning algorithms. Additionally, it sought to identify factors using traditional statistical techniques and compare the outcomes between the two methods. Furthermore, this study aimed to highlight the effects of socioeconomic factors, health behavior, and residential factors, which may be obscured by physical and mental health variables, using a hierarchical method. As psychological factors are strongly correlated with suicidal ideation, the significance of other factors diminishes when all the factors in a model are included simultaneously. This mechanism also works in machine learning algorithms. Therefore, we conducted hierarchical modeling using three separate models.

## Materials and methods

### Study data

#### Data sources

This study utilized 2018 data from the Korea Health Panel (KHP), a dataset investigated and managed by the National Health Insurance Services (NHIS) and the Korea Institute for Health and Social Affairs. The KHP provides reliable and representative data due to several key characteristics. First, samples were collected using a two-stage stratified random cluster sampling method based on the nationwide Population and Housing Census. Additionally, trained investigators from the KHP conducted face-to-face interviews with the participants and utilized supplementary tools, such as a household ledger, health diary, or medical care receipts, to minimize information loss and recall bias. The total sample size of the KHP in 2018 was 17,008 individuals. After excluding non-responses and missing values, the remaining sample comprised 12,698 individuals. Among them, 7,170 were individuals aged 50 or older. For analysis of the machine learning model, the ratio of the presence or absence of suicidal thoughts was adjusted to 1:1 using random under sampling (RUS). This was done to prevent overfitting errors caused by imbalanced data, resulting in a reduced sample size of 340 participants. This study was permitted by the institutional review board of Yonsei University (approval number: 1041849-202310-SB-200-01).

#### Conceptual framework

**Fig 1:**
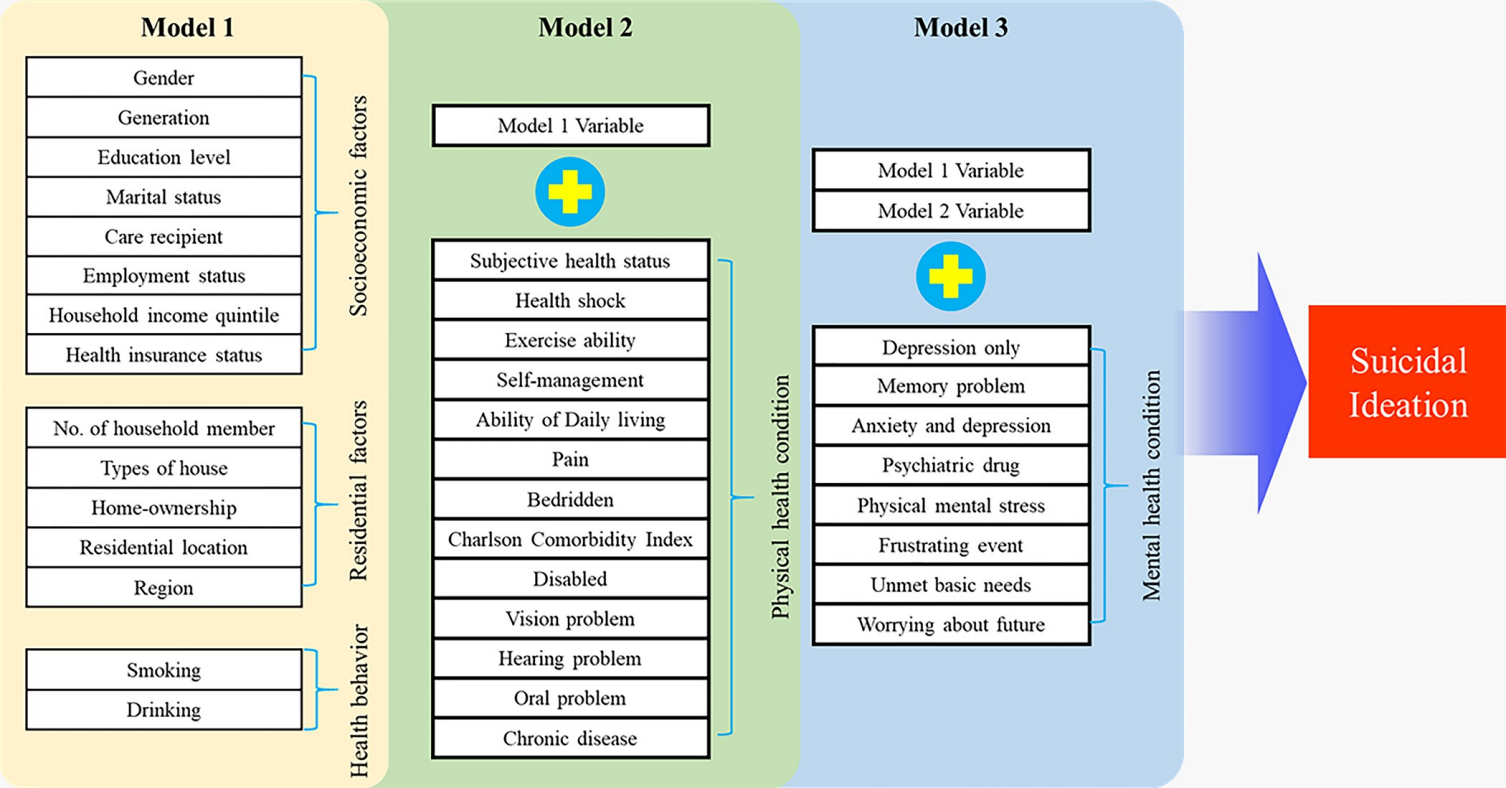
Conceptual framework.

The conceptual framework for this study is depicted in Fig 1. This framework suggests that various factors—including sociodemographic, health behaviors, residential characteristics, and physical and mental health—can predict the occurrence of suicidal ideation. A detailed explanation of these variables is provided in the following section.

#### Outcome variables

The dependent variable in this study was suicidal ideation, as defined by the question “Have you ever thought you wanted to die, in the past year?” If the response to the question was “Yes,” it was coded as 1; otherwise, it was coded as 0.

#### Independent variables

The advantage of machine learning techniques is that there is no limit to the input variables, and all variables provided by the data source can be input; however, conclusions can only be drawn through the given data values. Machine learning does not comprehend the relationships between variables in a human interpretive sense; rather, it interprets these relationships solely through numerical patterns. Therefore, this study included variables from existing studies with theoretical backgrounds to provide logical evidence [6, 9, 12, 13, 17]. Additionally, we adjusted the values of the variables for use in hierarchical multiple logistic regression (HMLR). Table 1 displays the computed codes of the variables, excluding the missing values.

**Table 1:** Independent variables and coding.

	Variables	Categories
Socioeconomic factors	Gender	Male (0); female (1)
	Generation	50~65 (3); 65~75 (4); ≥76 (5)
	Education level	≥College (0); Middle school (1); High school (2)
	Marital status	Married (0); Single (1)
	Care recipient	No (0); Yes (1)
	Employment status	Employed (0); Self-employed (1); Unpaid family workers (2); Unemployed (3)
	Household income quintile	1 (Poor) ~5 (Rich)
	Health insurance status	National health insurance (0); Medical aid (1)
Health behavior	Smoking	No (0); Yes (1)
	Drinking	No (0); Yes (1)
Residential factors	Household size	1~ more than 4
	Types of house	Single family house (0); Multi-family house (1); Row-house (2); Rental apartment (3); Studio (4)
	Home-ownership	Home owners (0); Rental for year units (1); Monthly rental (2); Provided housing* (3)
	Residential location	Ground (0); Basement/Rooftop (1)
	Region	Capital Seoul (0); Gyeonggi-do Province (1); Metropolitan city (2); Province (3)
Physical health condition	Subjective health status	Good (0); Normal (1); Bad (2)
	Health shock	More than 7 days of hospitalization (1); Otherwise (0)
	Exercise ability	Good (0); Mild (1); Severe (2)
	Self-management	Good (0); Mild (1); Severe (2)
	Ability of Daily living	Good (0); Mild (1); Severe (2)
	Pain	Good (0); Mild (1); Severe (2)
	Bedridden	Good (0); Mild (1); Severe (2)
	Charlson Comorbidity Index	0; 1; ≥2
	Disabled	No (0); Yes (1)
	Vision problem	Good (0); Mild (1); Severe (2)
	Hearing problem	Good (0); Mild (1); Severe (2)
	Oral problem	Good (0); Mild (1); Severe (2)
	Chronic disease**	KCD code
Mental health condition	Depression only	No (0); Yes (1)
	Memory problem	No (0); Yes (1)
	Anxiety or depression	Good (0); Mild (1); Severe (2)
	Psychiatric drug	No (0); Yes (1)
	Physical mental stress	No (0); Sometimes (1); Often (2)
	Frustrating event	No (0); Yes (1)
	Unmet basic needs	No (0); Yes (1)
	Worring about future	No (0); Sometimes (1); Often (2)

Note

*includes government housing and corporate housing from company

**Only was input in machine learning procedure

Table 1 shows the independent variables included in this study, including sociodemographic factors, health behavior, residential, and physical and mental health factors. To address the impact of chronic diseases, we employed the Charlson Comorbidity Index (CCI) using traditional statistical techniques. Meanwhile, in our machine learning analyses, we utilized raw Korean version of the International Classification of Diseases (KCD) codes because there are no variable limitations and there is less need for manipulation of input variables. Health shock was defined as hospitalization for more than 7 days, according to the methods described by Jung et al. [25] and Nguyen et al. [26].

### Analysis

#### Hierarchical modeling

This study involved the construction of Model 1, which combines socioeconomic, residential, and health behavior factors; Model 2, an extension of Model 1 with the inclusion of physical health factors; and Model 3, further augmented with the addition of mental health variables to Model 2. Hierarchical machine learning modeling and HMLR analysis were conducted to reveal the effects of socioeconomic and residential factors more efficiently, considering the significant hierarchical relations of psychological factors. Data preprocessing, descriptive statistics, and regression analyses were performed using Stata version 18. Machine learning modeling and visual score graphs were performed using Python (version 3.9.10).

#### Hierarchical machine learning modeling

Machine-learning modeling is a method in which a machine autonomously learns from data and selects a model that optimally predicts outcomes. During the machine learning process, the predictive performance is assessed by iteratively adding and removing variables from the model. In this iterative process, the feature importance is calculated by evaluating whether a specific variable significantly contributes to reducing prediction errors when added or removed from the model [25]. Machine-learning methods are broadly categorized into supervised learning, in which researchers specify variables, and unsupervised learning, in which only data are provided without predefined variables. Therefore, we hierarchically input variables into the model based on their influence, as identified in prior research. Models 1, 2, and 3 were constructed using the researcher’s prior knowledge, and the performance of each model was evaluated at every stage. We refer to this as hierarchical machine learning modeling.

Specifically, we developed several models to predict the dependent variable using logistic regression, gradient boosting, naive bayes, K-nearest neighbors (KNN), support vector machine, and deep neural network, and compared the performance of each model. The metrics of sensitivity, specificity, accuracy, and area under the receiver’s operating characteristics curve (AUC) were used to demonstrate a comprehensive and precise evaluation. To prepare the data for machine-learning analysis, we performed a data split, allocating 75% of the total data as training data and reserving the remaining 25% as test data. Additionally, we implemented a RUS procedure on the training data at a 1:1 ratio to mitigate overfitting errors in the unbalanced data. Machine learning becomes inefficient when the distribution of the binary dependent variable values is highly imbalanced (26). Therefore, this study addresses this imbalance by employing RUS to align the levels of individuals with and without suicidal ideation in a training dataset.

**Fig 2:**
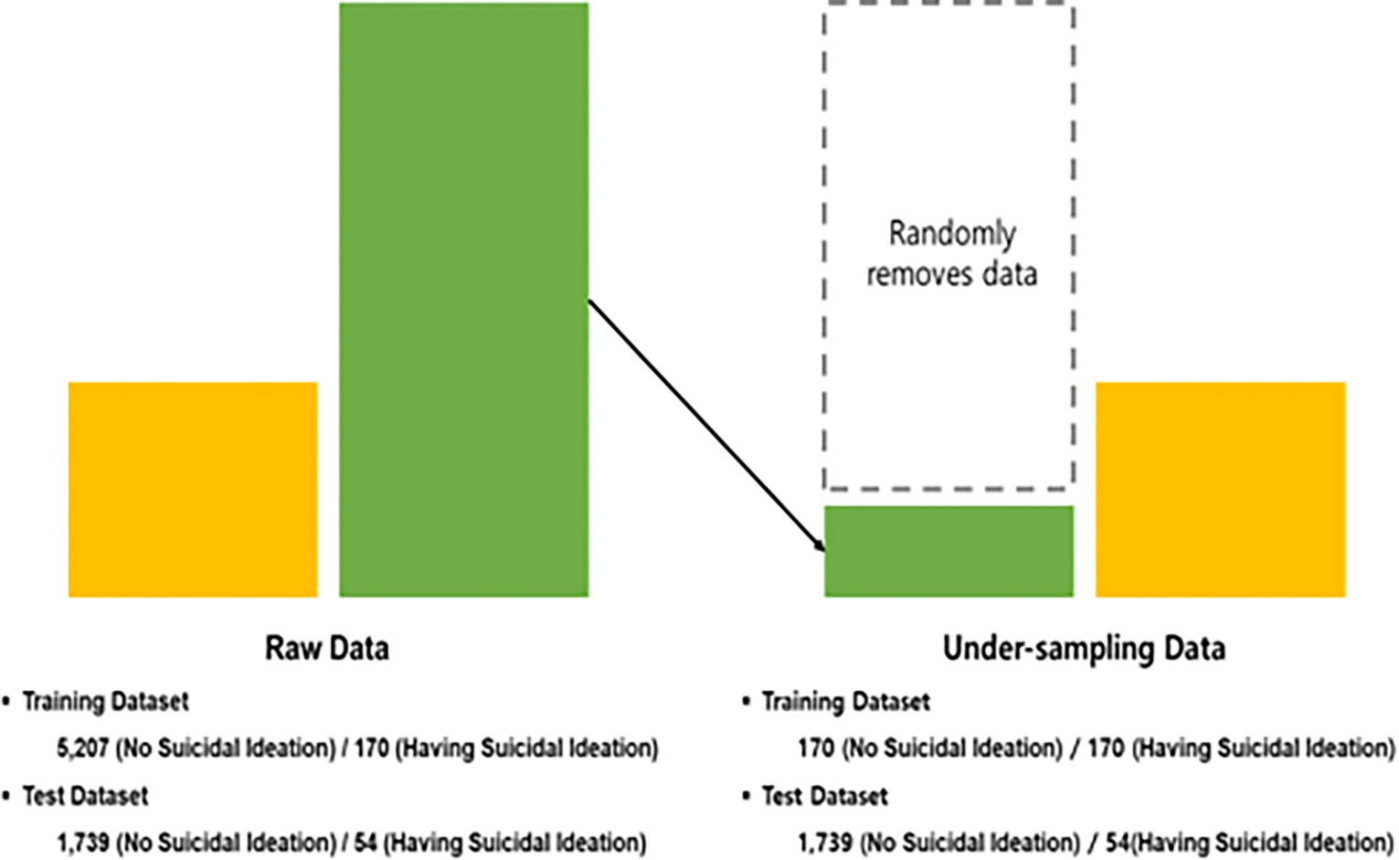
The process of random under sampling.

The RUS process is illustrated in Fig 2. The raw dataset comprises 7,170 older adults. Although we did not conduct machine learning with this dataset, it was divided into a training dataset of 5,377 individuals (5,207 without and 170 with suicidal ideation) and a test dataset of 1,793 individuals (1,739 without and 54 with suicidal ideation) if machine learning was applied without using RUS. After implementing the RUS, the number of individuals without suicidal ideation in the training dataset was balanced with the number of individuals with suicidal ideation, resulting in 340 individuals (170 with and 170 without suicidal ideation). The test dataset consisted of 1,793 individuals, with 1,739 without and 54 with suicidal ideation.

#### Hierarchical multiple logistic regression analysis

HMLR is a well-known model that goes one step further than the multiple regression model, which measures the relationship between various independent and binary dependent variables [27, 28]. The hierarchical regression model considers the systematic order and hierarchy in the process of the independent variables affecting the dependent variable. In this process, the researcher can check the adjusted coefficient whenever a new independent variable is added, better understand the relationships among the independent variables, and effectively comprehend the magnitude of the influence of a specific independent variable on the binary dependent variable [28]. The most significant advantage of a hierarchical regression model is that it expands when new independent variables are discovered in the traditional model. Hierarchical regression can reveal the shortcomings of the conventional model and show how the model improves the estimation precision by adding new predictors.

## Results

### Descriptive analyses

The Chi-square test results examining the relationship between the general characteristics of middle-aged and elderly individuals and each dependent variable are detailed in the S1 Appendix. In the overall sample, significant differences were observed in variables related to suicidal thoughts, excluding three variables: residential location (basement, rooftop, and above ground), region, and smoking status. After under sampling, significant differences persisted in all variables, except for house type, residential location, region, CCI, and smoking status. The interpretation and consideration of the aforementioned results are discussed in subsequent sections.

### Results of the prediction model for suicidal ideation

The results of the analysis using the six machine learning algorithms for Models 1–3, predicting suicidal thoughts in middle-aged and older individuals, are presented in Table 2. All metric values of sensitivity, specificity, accuracy, and AUC increased from Model 1 to Model 3, and among the six machine learning algorithms, logistic regression and gradient boosting consistently showed the highest performance.

**Table 2:** Comparison of performance of 6 machine learning algorithm-based prediction models.

	**Model 1 Prediction Performances**			
	**Sensitivity**	**Specificity**	**Accuracy**	**AUC**
Logistic Regression	0.721	0.537	0.716	0.629
Gradient Boosting	0.791	0.407	0.78	0.599
Naive Bayse	0.819	0.425	0.807	0.622
k-Nearest Neighbor	0.847	0.296	0.83	0.571
Support Vector Machine	0.878	0.333	0.861	0.605
Deep Neural Network	0.683	0.462	0.676	0.573
	**Model 2 Prediction Performances**			
Logistic Regression	0.707	0.740	0.708	0.724
Gradient Boosting	0.659	0.796	0.663	0.727
Naive Bayse	0.782	0.518	0.774	0.650
k-Nearest Neighbor	0.861	0.37	0.846	0.615
Support Vector Machine	0.705	0.666	0.703	0.685
Deep Neural Network	0.635	0.777	0.64	0.706
	**Model 3 Prediction Performances**			
Logistic Regression	0.836	0.851	0.836	0.843
Gradient Boosting	0.868	0.814	0.867	0.841
Naive Bayse	0.794	0.851	0.795	0.822
k-Nearest Neighbor	0.917	0.537	0.905	0.727
Support Vector Machine	0.849	0.814	0.848	0.832
Deep Neural Network	0.834	0.851	0.834	0.843

When initially observed, the gradient boosting- and logistic regression-based prediction models in Model 1 showed fairly good performance, with an AUC of 0.6 or higher. However, the KNN-based prediction model in Model 1 showed high prediction accuracy in terms of sensitivity (0.847) and accuracy (0.83), but very low prediction accuracy in terms of specificity (0.296). This indicates that the model is relatively overfitted to accurately predict only positive predictions. As additional learning variables were added from Models 1 to 2 and Models 2 to 3, the accuracy performance tended to gradually increase. Finally, majority of the prediction models in Model 3 showed very good accuracies above 0.80 for all indices, sensitivity, specificity, accuracy, and AUC.

### Feature importance of suicidal ideation

**Fig 3:**
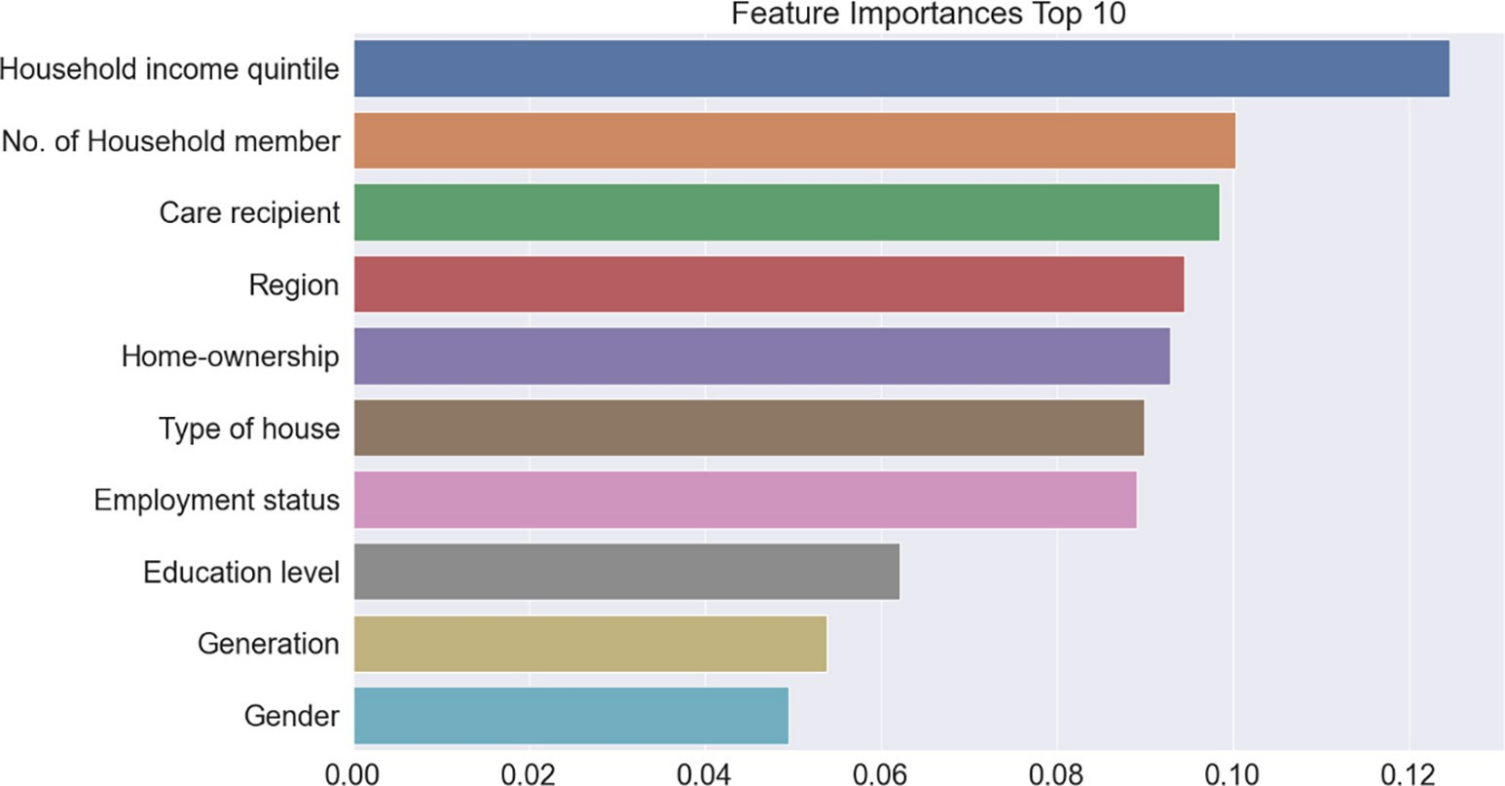
Feature importance extracted during Model 1 learning process.

**Fig 4:**
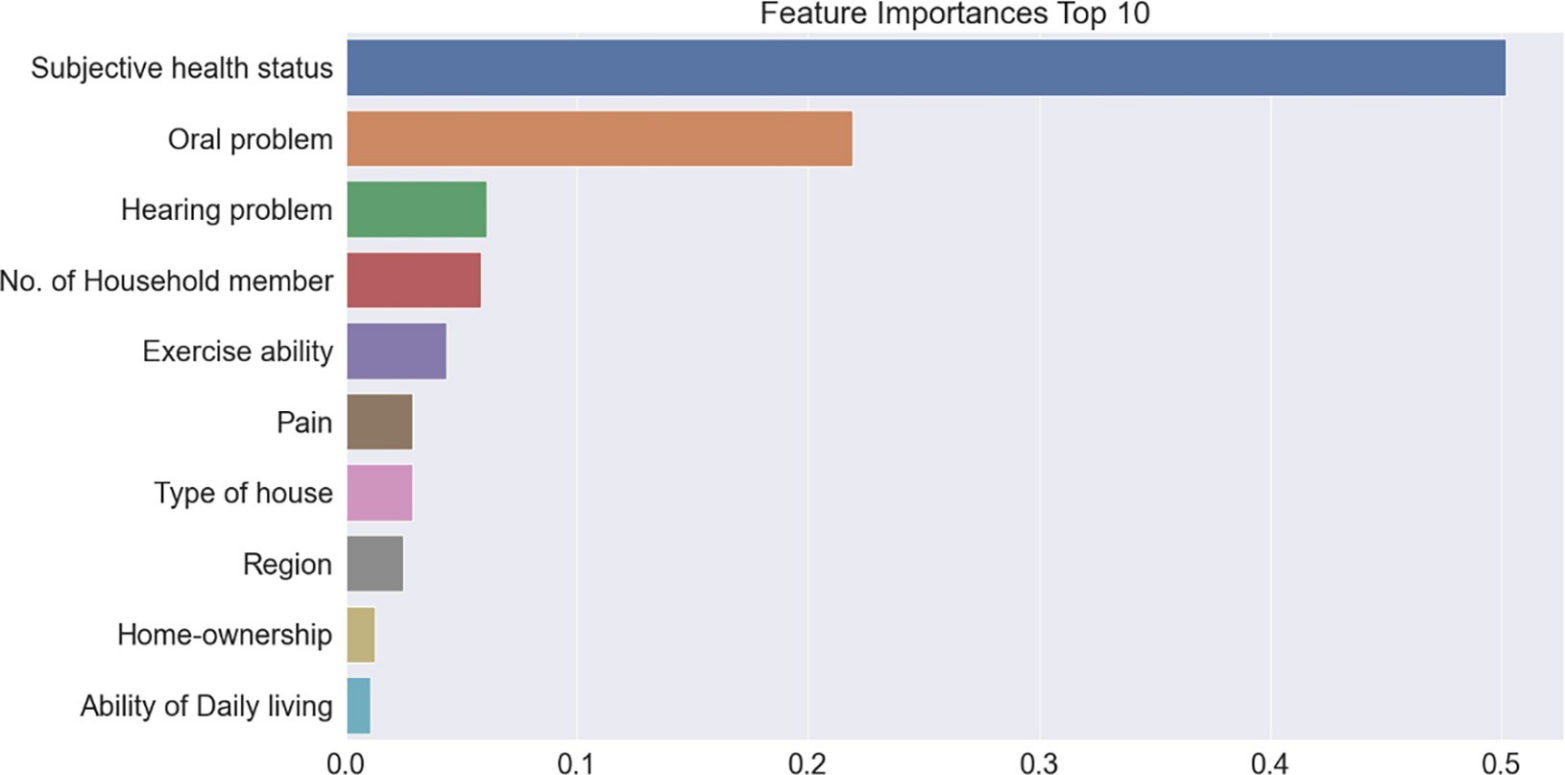
Feature importance extracted during Model 2 learning process.

**Fig 5:**
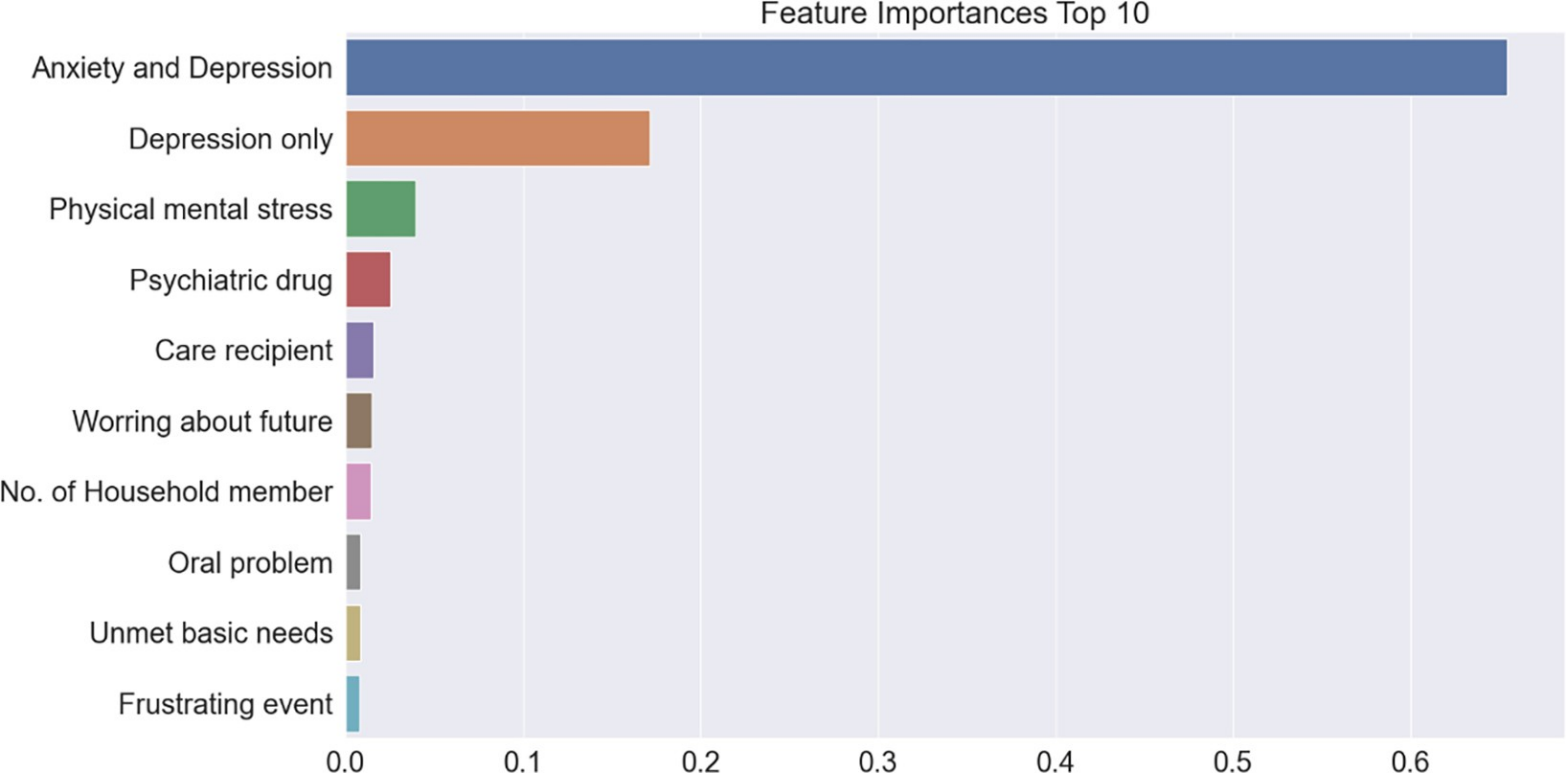
Feature importance extracted during Model 3 learning process.

Figs 3–5 present the top 10 feature importance variables at each stage of the models calculated by gradient boosting, which exhibited the highest accuracy. In Model 1, the most prominent feature was the household income quintile, followed by the number of household members, care recipients, region, home ownership, type of house, employment status, education level, generation, and gender. In Model 2, the most influential variable was subjective health status, followed by oral health and hearing problems, number of household members, exercise ability, pain, type of house, region, home ownership, and daily living ability. In Model 3, anxiety or depression was the most important factor, followed by depression alone, physical and mental stress, psychiatric drug use, care recipients, worrying about the future, number of household members, oral problems, unmet basic needs, and frustrating events.

### Results of hierarchical multiple regression analysis

From the results of the HMLR analysis, the pseudo-R^2^ significantly improved from Models 1 to 3 (Table 3). Specifically, the pseudo-R^2^ values were 0.06, 0.16, and 0.41 for Models 1, 2, and 3, respectively. In Model 1, a lower education level was associated with a higher likelihood of suicidal thoughts. Specifically, middle school education showed odds of 1.27 [0.74–2.19], and high school education had odds of 1.49 [0.89–2.49]. Similarly, the lower the income levels, the higher the odds of suicidal thoughts, with quintile 1 (Poor) at 2.47 odds [1.30–4.71], quintile 2 at 2.35 odds [1.31–4.20], and quintile 3 at 1.78 odds [0.99–3.19]. Additionally, the medical aid recipients were more likely to have suicidal thoughts (1.43 odds [0.93–2.22]). Regarding housing types, individuals who lived in multi-family houses (1.77 odds [1.15–2.72]), or commercial buildings (2.22 odds [1.14–4.31]) were more likely to think about suicide than those who lived in single-family houses. Furthermore, in terms of home ownership, individuals with a dwelling house with a rental for a year unit showed 1.65 odds [1.00–2.70], and those with monthly rental had 2.11 odds [1.42–3.14], both indicating a higher likelihood of suicidal thoughts than that in homeowners. Lastly, individuals with care recipients exhibited the highest odds ratio 3.58 [2.49–5.17].

**Table 3:** Results of hierarchical multiple regression analysis.

Variables	Categories	Suicidal thinking		
		Model1	Model2	Model3
Gender (Male)	Female	1.23[0.86~1.77]	1.16[0.79~1.71]	0.90[0.55~1.33]
Generation (50~65)	65~75	1.02[0.69~1.50]	0.81[0.54~1.21]	1.14[0.75~1.88]
	≥76	1.02[0.65~1.60]	0.59**[0.36~0.95]	0.99[0.68~2.0]
Education level (≥ College)	Middle school	1.27[0.74~2.19]	0.86[0.49~1.51]	1.29[0.69~2.51]
	High school	1.49*[0.89~2.49]	1.20[0.70~2.06]	1.42[0.78~2.65]
Marital status (Married)	Single	0.86[0.53~1.41]	0.83[0.50~1.37]	0.99[0.57~1.75]
Employment status(Employed)	Self-employed	0.85[0.52~1.38]	0.74[0.45~1.22]	0.63[0.35~1.09]
	Unpaid family workers	1.21[0.58~2.53]	0.96[0.45~2.03]	0.94[0.39~2.24]
	Unemployed	1.04[0.71~1.51]	0.77[0.52~1.14]	0.71[0.48~1.19]
Household income quintile (Richest)	1 (Poor)	2.47***[1.30~4.71]	1.83[0.94~3.56]	1.61[0.70~3.05]
	2	2.35***[1.31~4.20]	1.86*[1.02~3.39]	1.36[0.69~2.66]
	3	1.78*[0.99~3.19]	1.60[0.88~2.92]	1.38[0.69~2.66]
	4	1.09[0.58~2.04]	1.01[0.54~1.91]	1.03[0.51~2.11]
Health insurance status (National health insurance)	Medical aid	1.43**[0.93~2.22]	1.11[0.70~1.77]	0.95[0.54~1.66]
No. of household member (≥ 4)	1	1.12[0.60~2.09]	1.05[0.55~2.00]	0.82[0.40~1.77]
	2	0.59**[0.38~0.92]	0.58**[0.36~0.92]	0.60[0.37~1.07]
	3	0.94[0.59~1.50]	0.93[0.57~1.50]	0.85[0.49~1.53]
Types of house (Single family house)	Multi-family house	1.77**[1.15~2.72]	1.58**[1.01~2.46]	1.35[0.76~2.16]
	Row-house	1.21[0.54~2.70]	1.12[0.48~2.60]	0.92[0.30~2.35]
	Rental apartment	1.14[0.79~1.63]	1.08[0.75~1.56]	0.99[0.65~1.53]
	Commercial building	2.22**[1.14~4.31]	2.04**[1.02~4.08]	2.46**[1.07~5.20]
Home-ownership (Home owners)	Rental for year units	1.65**[1.00~2.70]	1.98***[1.18~3.30]	1.71*[0.95~3.14]
	Monthly rental	2.11***[1.42~3.14]	1.92***[1.26~2.93]	1.32[0.81~2.23]
	Provided housing	1.06[0.66~1.68]	0.93[0.58~1.51]	1.05[0.62~1.88]
Residential location (Ground)	Basement, Rooftop	0.93[0.35~2.47]	0.86[0.30~2.43]	0.48[0.14~1.79]
Region (Capital Seoul)	Gyeonggi-do Province	0.89[0.55~1.46]	0.86[0.52~1.42]	1.23[0.70~2.30]
	metropolitan	0.70[0.44~1.12]	0.67[0.41~1.09]	1.00[0.61~1.90]
	Province	0.81[0.51~1.28]	0.73[0.45~1.18]	1.04[0.63~1.96]
Care recipient (No)	Yes	3.58***[2.49~5.17]	1.95***[1.26~3.02]	2.49***[1.50~4.14]
Smoking (No)	Yes	1.40[0.91~2.14]	1.29[0.83~2.02]	1.09[0.66~1.82]
Drinking (No)	Yes	0.91[0.67~1.23]	1.01[0.73~1.38]	1.20[0.82~1.77]
Health shock (No)	Yes		1.32[0.90~1.94]	1.14[0.72~1.82]
Subjective health status (Good)	Normal		1.37[0.84~2.25]	0.73[0.41~1.27]
	Bad		3.28***[1.93~5.59]	0.91[0.49~1.68]
Exercise ability (No)	Mild		1.65**[1.07~2.56]	2.19***[1.32~3.62]
	Severe		0.47[0.13~1.71]	0.63[0.13~3.11]
Self-management (No)	Mild		0.85[0.51~1.41]	0.90[0.49~1.62]
	Severe		1.17[0.36~3.78]	1.64[0.40~6.73]
Ability of Daily living (No)	Mild		0.97[0.61~1.56]	0.58[0.33~1.00]
	Severe		2.49[0.82~7.53]	0.68[0.19~2.42]
Pain (No)	Mild		1.30[0.88~1.93]	0.76[0.48~1.20]
	Severe		1.35[0.66~2.75]	0.38**[0.16~0.90]
CCI (0)	1		0.82[0.58~1.16]	0.97[0.65~1.45]
	≥2		0.73[0.49~1.10]	0.77[0.48~1.23]
Disabled (No)	Yes		0.99[0.67~1.48]	1.00[0.63~1.59]
Vision problem (No)	Mild		1.4**[0.99~1.98]	1.19[0.79~1.78]
	Severe		1.21[0.68~2.12]	0.85[0.43~1.68]
Hearing problem (No)	Mild		1.80***[1.27~2.55]	1.61**[1.07~2.41]
	Severe		1.17[0.63~2.17]	1.24[0.62~2.46]
Oral problem (No)	Mild		1.67**[1.06~2.64]	1.63**[0.98~2.71]
	Severe		2.44***[1.57~3.79]	1.83**[1.12~2.99]
Memory problem (No)	Yes		0.92[0.57~1.50]	0.48**[0.26~0.86]
Bed-ridden (No)	Yes		1.91***[1.17~3.13]	1.85**[1.01~3.35]
Depression only (No)	Yes			6.98***[4.80~10.15]
Anxiety and depression (No)	Mild			3.62***[2.44~5.38]
	Severe			12.93***[4.14~40.34]
Psychiatric drug (No)	Yes			1.52**[1.02~2.27]
Physical mental stress (No)	Sometimes			2.10***[1.15~3.82]
	Often			4.13***[2.05~8.32]
Frustrating event (No)	Yes			1.78**[1.14~2.81]
Unmet basic needs (No)	Yes			1.46[0.92~2.32]
Worrying about future (No)	Sometimes			1.58*[0.95~2.63]
	Often			2.68***[1.39~5.15]
Constants		0.01***[0.00~0.02]	0.00***[0.00~0.01]	0.00***[0.00~0.00]
Pseudo R2		0.06***	0.16***	0.41***
Number of observation		7,170	7,170	7,170

In Model 2, which incorporated physical health condition factors, the odds ratios for many socioeconomic factors, including age, educational level, employment status, household income quintile, and health insurance status, were significantly reduced. However, most health behaviors and residential factors either remained unchanged or showed significant differences. Among the physical health variables, those reporting bad in subjective health status (3.28 odds [1.93–5.59]), oral problem (mild, severe; 1.67 odds [1.06–2.64], 2.44 odds [1.57–3.79], respectively), mild hearing problem (1.80 odds [1.27–2.55]), mild exercise ability (1.65 odds [1.07–2.56]), mild vision problem (1.4 odds [0.99–1.98]), and bedridden (1.91 odds [1.17–3.13]) were statistically significant.

Model 3, which included all factor variables, improved substantially in pseudo R^2^ and majority mental health conditions were statistically significant. Owing to the effects of physical and mental health variables, all socioeconomic factors become insignificant. Moreover, the number of household member (2-person household; 0.60 odds [0.37–1.07]), multi-family house (1.35 odds [0.76–2.16]), households with rental for year unit (1.32 odds [0.81–2.23]), bad condition in subjective health status (0.91 odds [0.49–1.68]), and mild vision problem (1.19 odds [0.79–1.78]) became insignificant, which is contrary to the results of Model 2. Meanwhile, severe pain (0.38 odds [0.16–0.90]) and memory problems (0.48 odds [0.26–0.86]) become significant. Furthermore, majority of the mental health variables, including anxiety and depression, depression alone, physical and mental stress, psychiatric drugs use, worrying about the future, and frustrating events, showed a high odds ratio compared to the other previous factors and were statistically significant. Especially the depression alone (6.98 odds [4.80–10.15]) and severe anxiety or depression (12.93 odds [4.14–40.34]) presented extraordinarily high odds ratios.

## Discussion

The results of the study indicated that the gradient boosting algorithm outperformed the other machine learning techniques. Furthermore, the household income quintile was the most important feature in Model 1, followed by subjective health status, oral health, and hearing problems in Model 2, and anxiety and depression in Model 3. These results correspond to those of the hierarchical logistic regression. The S1 Appendix displays the basic statistical results for the samples, illustrating the distribution of suicidal ideation across the independent variables. A comparison between the total sample and RUS offers insights into the RUS method, which equalizes the sample size for both those with and without suicidal thinking. Unlike propensity score matching, which adjusts the control group based on the treatment group, RUS randomly reduces the control group in the total sample to achieve proportionality. For example, the gender composition of individuals who reported no suicidal ideation in RUS closely resembled that of the total sample rather than aligning with those who reported affirmative responses in RUS. Had the study employed propensity score matching, none of the chi-squared results would have exhibited significance.

Table 2 presents the test results for the six machine learning prediction models. Evaluation metrics, including sensitivity, specificity, accuracy, and AUC, was employed to assess the performance of these models. Each metric is usually categorized as follows: < 0.6 is classified as poor, 0.6–0.69 as fair, 0.7–0.79 as good, 0.8–0.89 as very good; and anything exceeding 0.9, excellent.

First, based on gradient boosting, which exhibited the highest prediction performance and the most crucial evaluation metric, AUC, Model 1 demonstrated the lowest prediction performance (ranked fair), while Model 3 presented the highest performance, ranking in the very good category. In the case of Model 1, although all accuracy performances tended to be lower than those of Models 2 and 3, they still had significant implications for health and social policy.

Whereas the physical and mental health conditions included in Models 2 and 3 are subjective elements that cannot be detected without individuals’ self-reports, socioeconomic and residential information is observable from the outside. The fundamental problem with suicide is its unpredictability. Most people with suicidal thoughts do not share their minds with others because of the seriousness of the issue. In many cases, they suddenly commit suicide without any signs or expressions [29]. This concealing tendency makes it difficult for the government to achieve its goal of suicide prevention. However, by using socioeconomic and residential information, organizations responsible for preventing suicides can focus on target groups and intervene collectively, thereby using resources efficiently.

Moreover, the high sensitivity and low specificity of Model 1 indicate that the machine effectively predicts and identifies individuals with suicidal ideation, but also frequently misclassifies individuals without suicidal thoughts to be having them. While some may perceive this as a limitation, from a positive standpoint, it underscores the potential of identifying individuals at high risk of suicide solely through socioeconomic and residential factors. The results with high sensitivity and low specificity are much better than those with low sensitivity and high specificity, considering the purpose of prediction, which is to prevent suicide.

After establishing the preceding prediction performance, we confirmed the feature importance of each hierarchical model using gradient boosting, which can prioritize health policies. First, gradient boosting captured the household income quintile as the most important feature of Model 1’s variable importance, and it was found that variables related to economic factors, such as region, home ownership, type of house, and employment status, also had a significant impact on suicidal thoughts. We also discovered an interesting finding: the care recipient was extracted as an important feature. While most studies have proven that caregivers are significantly vulnerable to suicidal thinking [30, 31], this study revealed an additional finding that care recipients are also more likely to consider suicide and that its importance is incomparable to that of other factors. Intuitively, it may seem that the recipients would have been satisfied when they received care, but this was not the case.

This can be interpreted as follows: first, suicidal ideation could be contagious among close relationships [32]. Second, the physical and mental health of the care recipients had already deteriorated; therefore, they would have considered suicide. Third, they might have feelings of guilt toward their caregivers because they show unwanted shameful situations and cannot help but always need the hands of the caregivers. Finally, they felt sorry for their families due to the costs spent on their livelihoods and paid caregivers. Therefore, it is necessary to establish psychological education programs for both caregivers and recipients.

In Model 2, oral health problems, hearing health problems, the number of household members, and exercise ability were the major factors affecting feature importance. It is well known that physical health affects suicidal ideation [33, 34]; thus, the highest priority seems to be the characteristics of middle-aged and older individuals. An interesting point is the oral health problems. A considerable number of studies have provided evidence that oral health is related to suicidal thoughts [35–37], but its importance has been underestimated compared to other variables, such as depression. This may be because oral health problems abandon the life-giving pleasure of enjoying good foods. Furthermore, we suggest another possible interpretation: people may unconsciously associate suicide with difficulty in eating because it is genetically linked to survival.

In Model 3, ‘anxiety or depression’ and ‘depression only’ stood out prominently in terms of feature importance. Unfortunately, KHP investigated ‘anxiety or depression’ not ‘anxiety only.’ Although both variables measure depression, the ’anxiety or depression’ variable includes an additional element of anxiety. Moreover, notable differences were observed in the values and distributions of the two variables. Therefore, we included both variables in the model. Anxiety and depression are closely related variables. Although depression has traditionally been considered a trigger and key factor in suicidal ideation, anxiety has received relatively less attention [12, 16, 38, 39]. Therefore, anxiety should be considered a significant variable.

The results of the HMLR analysis (Table 3) were similar to those of the hierarchical stepwise feature importance analysis. The regression coefficient values and significance levels of the variables that appeared to be important for the feature importance of each model were the highest. However, while machine learning cannot articulate the vectors of dependent variables, the HMLR analysis can.

In Model 1 of the HMLR, individuals below the median income level, medical aid recipients, tenants in multifamily houses, and those residing in commercial buildings, including all types of tenants (lump-sum deposit and monthly rent tenants), were more likely to experience suicidal ideation. However, in Model 3, in which physical and mental health variables were added, most of the socioeconomic factors became insignificant. This seems to be because income- and residence-related variables are correlated with physical and mental health factors.

Interpreting the results of HMLR with specific reference to previous research, we first address the gender aspect. There exists a well-known paradox regarding suicide rates: although the male suicide rate is typically higher than that of females, females are more likely to consider suicide [40]. Existing studies have attributed this paradox to natural gender differences, suggesting that males tend to be impulsive with high execution abilities, whereas females are more sensitive and prone to overthinking [41, 42]. In model 1 of this study, females displayed 1.23 times higher odds of experiencing suicidal thoughts compared to males, although this disparity was not statistically significant. Moreover, in the model 3, the odds decreased to 0.9. We cautiously suggest that a confounding factor may influence the relationship between gender characteristics and suicidal ideation. Additionally, it’s noteworthy that findings indicating higher rates of suicidal thoughts among women compared to men are not consistently observed. For example, Han and Lee [12] found no significant difference between genders.

Han and Lee [12] utilized a similar model to this study in that it employed a hierarchical approach and included comparable socioeconomic and health behavior variables. They reported insignificance for gender, age, educational level, employment status, marital status, smoking, and drinking, which aligns with the findings of this research. However, other studies reported varying results. For instance, Pompili et al. [43] discovered that highly educated individuals may resort to suicide following experiences of failure or public shame, whereas YEN [44] reported that the lower the level of education, the more suicidal thoughts occur. Qingsong et al. [45] found that employment status significantly influenced suicidal thoughts in the USA and Europe but not in Taiwan. Meanwhile, Yeong et al. [46] reported that low socioeconomic status heightens the risk of suicidal ideation among the elderly. Additionally, regarding marital status, Erwin et al. [47] discovered that having a partner had no significant effect, aligning with the results of HMLR, and additionally revealed that experiencing the loss of a partner had an impact.

The results of physical health in HMLR also presented both similarities and differences compared to existing literature. Notably, all physical health variables utilized in this study have been previously proven as factors associated with suicidal ideation: subjective health [48], exercise ability [49], self-management [50], daily living ability [51], pain [52], CCI [53, 54], disability [55–57], vision problem [58–60], hearing problem [61–63], oral problem [64, 65], memory problem [66], bedridden [67]. However, this study showed different results in that self-management, ability of daily living, CCI, and disability were not significant. These disparities may stem from national characteristics, data sources, and different variable composition within models.

On the other hand, both the results of feature importance and HMLR in this study corroborate findings from previous research indicating that mental health is the most significant factor in predicting suicidal thoughts. A wealth of literature has repeatedly confirmed similar results to those of this study [68–78]. All mental health variables included in the study models exerted a significant influence on suicidal ideation, except for unmet basic needs. The effects of these mental health variables can be found elsewhere, such as, depression [68–70], anxiety [71, 72], psychiatric drugs [73], physical mental stress [74, 75], frustrating events [76, 77], and unmet basic needs [78].

Although this study’s findings on factors contributing to suicidal ideation are substantial, the main finding is residential factors. It is well known that residential elements, including house type, influence overall mental health [79]. First, the deteriorated physical environments of majority of commercial buildings, such as poor hygiene, small rooms that cause a sense of confinement, and dirty building walls, yield unpleasant feelings [79]. Second, due to architectural design, substantial noise stress exists in collective forms of housing, including multifamily houses and commercial buildings. In South Korea, the prevalence of noise-induced stress and interpersonal conflict among residents has become a conspicuous societal concern. The severity of these noise-related disputes has, in some instances, reached a critical threshold, resulting in instances of neighboring homicides [80]. Third, the compact dimensions of commercial building rooms render residents unsuitable for hosting visits by acquaintances, relatives, or separated family members, thereby amplifying the likelihood of social isolation.

However, it is not feasible for the government to provide new housing or refurbish existing accommodations. The optimal course of action for the government involves broadening the living environment to allow social activities. Extending living areas, communal interaction, and readily available counseling and educational resources are needed, which can be provided by institutions or organizations, such as senior centers, nursing homes, or public clubs. Policy frameworks should not be exclusively confined to the public sector; there is a pressing need to incentivize initiatives within the private sector, including religious institutions and community organizations.

Residential elements represent socioeconomic aspects. First, home ownership represents financial stability and a proactive lifestyle. In the absence of home ownership, residing in another person’s dwelling may lead to relinquishing autonomy over one’s life and living space to others, potentially causing psychological distress and melancholy. For several years, housing crises related to skyrocketing real estate prices have been pervasive in South Korea [81, 82]. Those who could not buy houses in this period had regrets and sadness because the increased price of the house made them believe that they could not own houses forever, even if they worked for several decades. Majority of these people were rental residents [82, 83].

Specifically, middle-aged and older individuals face uncertainty regarding the duration of their employment and contend with income instability, thereby necessitating constant concern about the prospect of eviction if they are unable to meet rental obligations. Fortunately, the Right to Renew House Lease Contracts, which guarantee a residence period of at least 2 years, was established by a legal amendment of the Housing Lease Protection Act 2020 in Korea [83]. Therefore, a follow-up study is needed to evaluate suicidal ideation among tenants, even after the establishment of the law.

This research makes a contribution to the existing literature by adopting a novel approach—machine learning algorithms. It offers a comprehensive explanation by comparing the outcomes of machine learning with those derived from conventional logistic regression. Furthermore, the study brings attention to a previously overlooked aspect: individuals who are care recipients and have low income, residing in vulnerable residential conditions, are susceptible to suicidal ideation. This finding addresses a gap in previous research. Despite these strengths, the study has notable limitations. The model 3 of HMLR includes an excessive number of variables, potentially leading to model complexity problem. While removing non-significant variables could be considered optimal, Table 3 is designed to showcase the hierarchical structure and facilitate comparison with the machine learning process. As a result, we present the naive results of the models without excluding any variables.

## Conclusion

This study identified factors that could affect suicidal ideation in middle-aged and older people in as many categories as possible and identified priorities through a combination of machine learning techniques and traditional statistical techniques. As a result of the analysis, anxiety and depression were highest in the most expanded final model, though, physical health factors also played an important role. A hierarchical approach revealed that variables related to care recipients and residential areas caused significant suicidal thoughts. Although it is not possible to dramatically change people’s socioeconomic levels and living patterns directly, culture and policies that can be integrated with the local community should be developed by understanding why people in this environment have suicidal ideation. About a few decades ago, there was a Korean proverb, ’A neighbor is better than a distant relative.’ Unfortunately, with the prevalence of apartment-style residential complexes and the emergence of individualism during the rapid urban development in Korea, public interactions among neighbors have substantially diminished. It is crucial to revive both the phrase and the cultural values associated with it.

## Supporting information

S1 Appendix Chi-square test results. (DOCX)

## References

[1] Ra CK, Cho Y. Differentiated effects of social participation components on suicidal ideation across age groups in South Korea. BMC public health. 2013;13:1–8. 24067075 10.1186/1471-2458-13-890 PMC3850940

[2] WHO. Suicide worldwide in 2019: global health estimates. 2021.

[3] OECD. Health at a Glance 2023: OECD Indicators. Paris; 2023.

[4] Suicide rates (indicator) [Internet]. 2024 [cited 23 Jan 2024].

[5] KFSP. WHITE PAPER ON SUICIDE PREVENTION. Seoul: Ministry of Health & Welfare; 2022.

[6] Yoon S, Cummings S. Factors protecting against suicidal ideation in south Korean community-dwelling older adults: a systematic literature review. Journal of Gerontological Social Work. 2019;62(3):279–305. doi: 10.1080/01634372.2018.1557310 30556492

[7] Jee Y, Kim K. Predictors of suicidal thoughts in high school in Korea. Asia-Pacific Journal of Multimedia Services Convergent with Art, Humanities, and Sociology. 2015;5:123–31.

[8] Crocker L, Clare L, Evans K. Giving up or finding a solution? The experience of attempted suicide in later life. Aging and Mental Health. 2006;10(6):638–47. doi: 10.1080/13607860600640905 17050093

[9] Kim S, Jang Y, Seo H. The influence of marital satisfaction on the suicidal ideation of the elderly: Focusing on mediating effect of depression. J Korean Gerontol Soc. 2011;31(2):305–19.

[10] McIntosh JL, Hubbard RW, Overholser JC, Santos JF. Elder suicide: Research, theory and treatment: American Psychological Association Washington, DC; 1994.

[11] Miller DLS S., Frederick L. Coolidge Jill. A comparison of suicidal thinking and reasons for living among younger and older adults. Death studies. 2001;25(4):357–65. doi: 10.1080/07481180126250 11803985

[12] Han S, Lee H-S. Factors associated with suicidal ideation: the role of context. Journal of Public Health. 2013;35(2):228–36. doi: 10.1093/pubmed/fds097 23239079

[13] Kim HS. A study on epistemology of Korean elder’s suicidal thought. Journal of the Korea Gerontological Society. 2002;22(1):159–72.

[14] Conwell Y. Suicide in later life: a review and recommendations for prevention. Suicide and Life-Threatening Behavior. 2001;31(Supplement to Issue 1):32–47. doi: 10.1521/suli.31.1.5.32.24221 11326758

[15] Park MK, Jun HJ. Predictors of Depressive Symptom Trajectories among Baby Boomers with Experience of Suicide Ideation. Korea Gerontological Society. 2014;34(4):877–96.

[16] Kim SM, Lee G. Risk Factors of Suicide Ideation in Younger-Old and Older-Old Persons: Using Data from the Korea Health Panel Survey. Journal of Korean Gerontological Nursing. 2020;22(4):281–90.

[17] Lee SW. A longitudinal study on predictors of suicide ideation in old people: using a panel logit model. Health and Social Welfare Review. 2017;31(3):191–229.

[18] Kim Y, Lee E, Joo H. Exploring a predictive variables for the university entrance through Korean-Type Early Decision Programs. Journal of educational studies. 2019;50(4):233–55.

[19] Lee G-S. Exploring the Predictive Variables of Government Statistical Indicators on Retail sales Using Machine Learning: Focusing on Pharmacy. Journal of Korean Society for Internet Information. 2022;23(3).

[20] Park S, Chung H. Exploring Variables Affecting Career Decision of Middle School Students: An Application of Machine Learning Approaches. Asian Journal of Education. 2020;21(3):727–53.

[21] Lee K, Kim Y-s. Exploring a Predictive Model for the Employment of Persons with Disabilities Utilizing a Random Forest method: Focusing on the Status and Quality of Employment. Disability & Employment. 2019;29(3):145–65.

[22] Ayodele TO. Machine learning overview. New Advances in Machine Learning. 2010;2(9–18):16.

[23] Diez Roux AV, Mair C. Neighborhoods and health. Annals of the New York academy of sciences. 2010;1186(1):125–45. doi: 10.1111/j.1749-6632.2009.05333.x 20201871

[24] Pickett KE, Pearl M. Multilevel analyses of neighbourhood socioeconomic context and health outcomes: a critical review. Journal of Epidemiology & Community Health. 2001;55(2):111–22. doi: 10.1136/jech.55.2.111 11154250 PMC1731829

[25] Jung H, Yang J, Kim E, Lee J, editors. The effect of mid-to-long-term hospitalization on the catastrophic health expenditure: focusing on the mediating effect of earned income loss. Healthcare; 2021: MDPI. 10.3390/healthcare9081013 PMC8391954 34442150

[26] Rose S. Robust machine learning variable importance analyses of medical conditions for health care spending. Health Services Research. 2018;53(5):3836–54. doi: 10.1111/1475-6773.12848 29527659 PMC6153184

[27] Mohammed R, Rawashdeh J, Abdullah M, editors. Machine learning with oversampling and undersampling techniques: overview study and experimental results. 2020 11th international conference on information and communication systems (ICICS); 2020: IEEE.

[28] Witte JS, Greenland S. Simulation study of hierarchical regression. Statistics in medicine. 1996;15(11):1161–70. doi: 10.1002/(SICI)1097-0258(19960615)15:11&lt;1161::AID-SIM221&gt;3.0.CO;2-7 8804145

[29] Wong GY, Mason WM. The hierarchical logistic regression model for multilevel analysis. Journal of the American Statistical Association. 1985;80(391):513–24.

[30] Blanchard M, Farber BA. “It is never okay to talk about suicide”: patients’ reasons for concealing suicidal ideation in psychotherapy. Psychotherapy Research. 2020;30(1):124–36. doi: 10.1080/10503307.2018.1543977 30409079

[31] Huang Y-C, Hsu S-T, Hung C-F, Wang L-J, Chong M-Y. Mental health of caregivers of individuals with disabilities: relation to suicidal ideation. Comprehensive psychiatry. 2018;81:22–7. doi: 10.1016/j.comppsych.2017.11.003 29195106

[32] Lavers G, Andriessen K, Krysinska K. A systematic review of the experiences and support needs of informal caregivers for people who have attempted suicide or experienced suicidal ideation. International journal of environmental research and public health. 2022;19(9):5181. doi: 10.3390/ijerph19095181 35564578 PMC9102006

[33] Mercy JA, Kresnow M-j, O’Carroll PW, Lee RK, Powell KE, Potter LB, et al. Is suicide contagious? A study of the relation between exposure to the suicidal behavior of others and nearly lethal suicide attempts. American journal of epidemiology. 2001;154(2):120–7. doi: 10.1093/aje/154.2.120 11447044

[34] Liew H-P. Depression and chronic illness: a test of competing hypotheses. Journal of Health Psychology. 2012;17(1):100–9. doi: 10.1177/1359105311409788 21712338

[35] Roy-Byrne PP, Davidson KW, Kessler RC, Asmundson GJ, Goodwin RD, Kubzansky L, et al. Anxiety disorders and comorbid medical illness. General hospital psychiatry. 2008;30(3):208–25. doi: 10.1016/j.genhosppsych.2007.12.006 18433653

[36] Kim YS, Kim H-N, Lee J-H, Kim S-Y, Jun E-J, Kim J-B. Association of stress, depression, and suicidal ideation with subjective oral health status and oral functions in Korean adults aged 35 years or more. BMC oral health. 2017;17:1–10. 10.1186/s12903-017-0391-4 PMC5481876 28645271

[37] Folayan MO, Tantawi ME, Oginni O, Oziegbe E, Mapayi B, Arowolo O, et al. Oral health practices and oral hygiene status as indicators of suicidal ideation among adolescents in Southwest Nigeria. PLoS One. 2021;16(2):e0247073. doi: 10.1371/journal.pone.0247073 33630858 PMC7906320

[38] Okoro CA, Strine TW, Eke PI, Dhingra SS, Balluz LS. The association between depression and anxiety and use of oral health services and tooth loss. Community dentistry and oral epidemiology. 2012;40(2):134–44. doi: 10.1111/j.1600-0528.2011.00637.x 21883356

[39] Carlson GA, Cantwell DP. Suicidal behavior and depression in children and adolescents. Journal of the American Academy of child Psychiatry. 1982;21(4):361–8. doi: 10.1016/s0002-7138(09)60939-0 7119310

[40] Koo CY, Kim JS, Yu J. A study on factors influencing elders’ suicidal ideation: Focused on comparison of gender differences. Journal of Korean Academy of Community Health Nursing. 2014;25(1):24–32.

[41] Canetto SS, Sakinofsky I. The gender paradox in suicide. Suicide and Life‐Threatening Behavior. 1998;28(1):1–23. 9560163

[42] Fung YL, Chan ZC. A systematic review of suicidal behaviour in old age: a gender perspective. Journal of Clinical Nursing. 2011;20(15‐16):2109–24. doi: 10.1111/j.1365-2702.2010.03649.x 21535272

[43] Pompili M, Vichi M, Qin P, Innamorati M, De Leo D, Girardi P. Does the level of education influence completed suicide? A nationwide register study. Journal of affective disorders. 2013;147(1–3):437–40. doi: 10.1016/j.jad.2012.08.046 23021379

[44] YEN YC, YANG MJ, YANG MS, LUNG FW, SHIH CH, HAHN CY, et al. Suicidal ideation and associated factors among community‐dwelling elders in Taiwan. Psychiatry and clinical neurosciences. 2005;59(4):365–71. doi: 10.1111/j.1440-1819.2005.01387.x 16048440

[45] Chang Q, Chan CH, Yip PS. A meta-analytic review on social relationships and suicidal ideation among older adults. Social science & medicine. 2017;191:65–76. doi: 10.1016/j.socscimed.2017.09.003 28910599

[46] Ju YJ, Park E-C, Han K-T, Choi JW, Kim JL, Cho KH, et al. Low socioeconomic status and suicidal ideation among elderly individuals. International psychogeriatrics. 2016;28(12):2055–66. doi: 10.1017/S1041610216001149 27456081

[47] Stolz E, Fux B, Mayerl H, Rásky É, Freidl W. Passive suicide ideation among older adults in Europe: a multilevel regression analysis of individual and societal determinants in 12 countries (SHARE). Journals of Gerontology Series B: Psychological Sciences and Social Sciences. 2016;71(5):947–58. doi: 10.1093/geronb/gbw041 27048569 PMC4982389

[48] Jung H, Jang JS. Predicting and Analyzing Suicidal Ideations in Middle and Older Adults: A Hybrid Study of Machine Learning and Traditional Statistical Methods. Korean Public Health Research. 2024;50(1):17–35.

[49] Fabiano N, Gupta A, Fiedorowicz JG, Firth J, Stubbs B, Vancampfort D, et al. The effect of exercise on suicidal ideation and behaviors: A systematic review and meta-analysis of randomized controlled trials. Journal of affective disorders. 2023;330:355–66. doi: 10.1016/j.jad.2023.02.071 36871911

[50] Song MK, Ward SE, Hladik GA, Bridgman JC, Gilet CA. Depressive symptom severity, contributing factors, and self‐management among chronic dialysis patients. Hemodialysis International. 2016;20(2):286–92. doi: 10.1111/hdi.12317 25998623 PMC4654980

[51] Dennis M, Baillon S, Brugha T, Lindesay J, Stewart R, Meltzer H. The influence of limitation in activity of daily living and physical health on suicidal ideation: results from a population survey of Great Britain. Social psychiatry and psychiatric epidemiology. 2009;44:608–13. doi: 10.1007/s00127-008-0474-2 19139796

[52] Smith MT, Edwards RR, Robinson RC, Dworkin RH. Suicidal ideation, plans, and attempts in chronic pain patients: factors associated with increased risk. Pain. 2004;111(1–2):201–8. doi: 10.1016/j.pain.2004.06.016 15327824

[53] Shiner B, Riblet N, Westgate CL, Young-Xu Y, Watts BV. Suicidal ideation is associated with all-cause mortality. Military medicine. 2016;181(9):1040–5. doi: 10.7205/MILMED-D-15-00496 27612350

[54] Raue PJ, Meyers BS, Rowe JL, Heo M, Bruce ML. Suicidal ideation among elderly homecare patients. International Journal of Geriatric Psychiatry: A journal of the psychiatry of late life and allied sciences. 2007;22(1):32–7. doi: 10.1002/gps.1649 16955449

[55] Russell D, Turner RJ, Joiner TE. Physical Disability and Suicidal Ideation: A Community-Based Study of Risk/Protective Factors for Suicidal Thoughts. Suicide and Life-Threatening Behavior. 2009;39(4). doi: 10.1521/suli.2009.39.4.440 19792985

[56] Jurišić B, Marušič A. Suicidal ideation and behavior and some psychological correlates in physically disabled motor-vehicle accident survivors. Crisis. 2009;30(1). doi: 10.1027/0227-5910.30.1.34 19261566

[57] McConnell D, Hahn L, Savage A, Dubé C, Park E. Suicidal ideation among adults with disability in Western Canada: a brief report. Community mental health journal. 2015;52:519–26. doi: 10.1007/s10597-015-9911-3 26202547

[58] Park J, Lee OE. Association between vision impairment and suicidal ideation among older adults: Results from National Survey on Drug Use and Health. Disability and Health Journal.13(4). doi: 10.1016/j.dhjo.2020.100939 32417146

[59] Lee OE-K, Park D, Park J. Association of vision impairment with suicide ideation, plans, and attempts among adults in the United States. Clinical Psychology. 2022;78(11). doi: 10.1002/jclp.23437 36017683 PMC9804446

[60] Rim TH, Lee CS, Lee SC, Chung B, Kim SS. Influence of visual acuity on suicidal ideation, suicide attempts and depression in South Korea. BMJ. 2015;99(8):1112–9. doi: 10.1136/bjophthalmol-2014-306518 25733526

[61] Akram B, Nawaz J, Rafi Z, Akram A. Social exclusion, mental health and suicidal ideation among adults with hearing loss: protective and risk factors. The Journal of the Pakistan Medical Association. 2018;68(3):388–93. 29540873

[62] Park J, Lee O, McKee M. Association between hearing loss and suicidal ideation among middle-aged and older adults. AGING & MENTAL HEALTH. 2022;26(6):1287–94. doi: 10.1080/13607863.2021.1919991 33979563

[63] Ahn JH, Yang JS, Jung J, Kang S, Jung SJ. Association between hearing loss and suicidal ideation: Discrepancy between pure tone audiometry and subjective hearing level. Journal of Affective Disorders. 2024;344:495–502. doi: 10.1016/j.jad.2023.10.063 37838269

[64] Kim YS, Kim H-N, Lee J-H, Kim S-Y, Jun E-J, Kim J-B. Association of stress, depression, and suicidal ideation with subjective oral health status and oral functions in Korean adults aged 35 years or more. BMC Oral Health. 2017;17(101). 10.1186/s12903-017-0391-4 PMC5481876 28645271

[65] Lee H-O, Kim S-M, Park J-Y. Association of Psychological Health and Perceived Oral Health in Elderly Individuals: Focusing on Depression, Stress, and Suicidal Ideation. Journal of dental hygiene science. 2017;17(5):398–404.

[66] Dabin Kim DK, Kounseok Lee, Nayeon Choi, Sungwon Roh,. Suicidal ideation among the elderly living in the community: Correlation with living arrangement, subjective memory complaints, and depression. Journal of Affective Disorders. 2022;298:160–5. doi: 10.1016/j.jad.2021.10.066 34710504

[67] Park E, Lee HY. Urban and rural differences in suicidal ideation and associated factors among older Koreans: Results from the Korean National Survey 2012–2013. Current Psychology. 2023;42:7002–11.

[68] Handley TE, Hiles SA, Inder KJ, Kay-Lambkin FJ, Kelly BJ, Lewin TJ, et al. Predictors of suicidal ideation in older people: a decision tree analysis. The American Journal of Geriatric Psychiatry. 2014;22(11):1325–35. doi: 10.1016/j.jagp.2013.05.009 24012228

[69] Guidry ET, Cukrowicz KC. Death ideation in older adults: psychological symptoms of depression, thwarted belongingness, and perceived burdensomeness. Aging & Mental Health. 2016;20(8):823–30. doi: 10.1080/13607863.2015.1040721 26035346

[70] Bhar SS, Brown GK. Treatment of depression and suicide in older adults. Cognitive and Behavioral Practice. 2012;19(1):116–25. 10.1016/j.cbpra.2010.10.002 PMC3289281 22383863

[71] Santini ZI, Koyanagi A, Tyrovolas S, Haro JM. The association of relationship quality and social networks with depression, anxiety, and suicidal ideation among older married adults: Findings from a cross-sectional analysis of the Irish Longitudinal Study on Ageing (TILDA). Journal of affective disorders. 2015;179:134–41. doi: 10.1016/j.jad.2015.03.015 25863909

[72] Wiebenga JX, Heering HD, Eikelenboom M, van Hemert AM, van Oppen P, Penninx BW. Associations of three major physiological stress systems with suicidal ideation and suicide attempts in patients with a depressive and/or anxiety disorder. Brain, behavior, and immunity. 2022;102:195–205. doi: 10.1016/j.bbi.2022.02.021 35202735

[73] Kiosses DN, Gross JJ, Banerjee S, Duberstein PR, Putrino D, Alexopoulos GS. Negative emotions and suicidal ideation during psychosocial treatments in older adults with major depression and cognitive impairment. The American Journal of Geriatric Psychiatry. 2017;25(6):620–9. doi: 10.1016/j.jagp.2017.01.011 28223082 PMC5429870

[74] Howarth EJ, O’Connor DB, Panagioti M, Hodkinson A, Wilding S, Johnson J. Are stressful life events prospectively associated with increased suicidal ideation and behaviour? A systematic review and meta-analysis. Journal of Affective Disorders. 2020;266:731–42. doi: 10.1016/j.jad.2020.01.171 32217256

[75] Shin Y-C, Lee D, Seol J, Lim S-W. What kind of stress is associated with depression, anxiety and suicidal ideation in Korean employees? Journal of Korean medical science. 2017;32(5):843. doi: 10.3346/jkms.2017.32.5.843 28378560 PMC5383619

[76] Rowe CA, Walker KL, Britton PC, Hirsch JK. The relationship between negative life events and suicidal behavior. Crisis. 2013. 10.1027/0227-5910/a000173 23261914

[77] Musetti A, Pingani L, Zagaria A, Uberti D, Meli S, Lenzo V, et al. Insecure adult attachment and reflective functioning as mechanisms of the relationship between traumatic life events and suicidal ideation: A path analysis. Frontiers in psychology. 2022;13:985148. doi: 10.3389/fpsyg.2022.985148 36248502 PMC9561888

[78] Tucker RP, Wingate LR. Basic need satisfaction and suicidal ideation: A self-determination perspective on interpersonal suicide risk and suicidal thinking. Archives of Suicide Research. 2014;18(3):282–94. doi: 10.1080/13811118.2013.824839 24810541

[79] Singh A, Daniel L, Baker E, Bentley R. Housing disadvantage and poor mental health: a systematic review. American journal of preventive medicine. 2019;57(2):262–72. doi: 10.1016/j.amepre.2019.03.018 31326010

[80] Kim E-s. Sound and the Korean public: sonic citizenship in the governance of apartment floor noise conflicts. Science as Culture. 2016;25(4):538–59.

[81] Park SM, Lee YJ, Park JH, Park JA, Lim JS, Kim HH, editors. Comparison of real estate index prediction models using machine learning and deep learning. Proceedings of the Korea Information Processing Society Conference; 2021: Korea Information Processing Society.

[82] Jang JS. A study on problems and its solutions of the Moon administration’s real estate policy. Seoul: Hanyang University; 2021.

[83] Chu S-H, Kim J-W. Some Issues on the Right to Renewal Lease under the revised Housing Lease Protection Act. Ewha law journal. 2020;25(1):111–57.

