# Supplementary material for: Constructing prediction models and analyzing factors in suicidal ideation using machine learning, focusing on the older population
Source: PLoS One. 2024 Jul 22;19(7):e0305777. doi: 10.1371/journal.pone.0305777 (PMC11262681; doi:10.1371/journal.pone.0305777)
Supplement: S1 Appendix — (DOCX) [file pone.0305777.s001.docx]

**Supporting information**

S1 Appendix 1. Chi-square test results

| Variables | Categories |  | Suicidal thinking | | | | |  |
| --- | --- | --- | --- | --- | --- | --- | --- | --- |
|  |  | Total sampling | | |  | Random under-sampling | |  |
|  |  | No | | Yes | Chi2  (P) | No | Yes | Chi2  (P) |
| Gender | Male | 3,056(44.0) | | 81(36.16) | 5.414  (0.02) | 83(48.82) | 57(33.5) | 8.208  (0.004) |
|  | Female | 3,890(56.0) | | 143(63.84) |  | 87(51.18) | 113(66.5) |  |
| Generation | 50~65 (3) | 3,558(51.22) | | 87(38.84) | 20.43  (0.000) | 90(52.94) | 60(35.29) | 11.36  (0.003) |
|  | 65~75 (4) | 1,982(28.53) | | 66(29.46) |  | 44(25.88) | 54(31.76) |  |
|  | $\geq$76 (5) | 1,406(20.24) | | 71(31.7) |  | 36(21.18) | 56(32.94) |  |
| Education level | Middle school | 1,203(17.32) | | 21(9.38) | 12.17  (0.002) | 24(14.12) | 14(8.23) | 7.433  (0.024) |
|  | High school | 3,604(51.89) | | 138(61.61) |  | 88(51.76) | 112(65.9) |  |
|  | $\geq$College | 2,139(30.79) | | 65(29.02) |  | 58(34.12) | 44(25.88) |  |
| Marital status | Married | 5,235(75.37) | | 132(58.93) | 31.15  (0.000) | 130(76.47) | 99(58.24) | 12.85  (0.000) |
|  | Single | 1,711(24.63) | | 92(41.07) |  | 40(23.53) | 71(41.76) |  |
| Caregiver | No | 6,395(92.1) | | 165(73.7) | 94.45  (0.000) | 160(94.1) | 120(70.6) | 32.38  (0.000) |
|  | Yes | 551(7.9) | | 59(26.3) |  | 10(5.9) | 50(29.4) |  |
| Employment status | Employed | 2,233(32.15) | | 55(24.55) | 19.24  (0.000) | 48(28.24) | 42(24.7) | 14.49  (0.002) |
|  | Self-employed | 1,281(18.44) | | 27(12.05) |  | 38(22.35) | 16(9.4) |  |
|  | Unpaid family worker | 352(5.07) | | 10(4.46) |  | 3(1.76) | 8(4.7) |  |
|  | Unemployed | 3,080(44.34) | | 132(58.93) |  | 81(47.65) | 104(61.2) |  |
| Household income quintile | 1 (Poor) | 1,506(21.68) | | 82(36.61) | 45.22  (0.000) | 30(17.65) | 65(38.24) | 23.15  (0.000) |
|  | 2 | 1,559(22.44) | | 62(27.68) |  | 43(25.29) | 43(25.29) |  |
|  | 3 | 1,291(18.59) | | 38(16.96) |  | 35(20.59) | 30(17.65) |  |
|  | 4 | 1,311(18.87) | | 23(10.27) |  | 29(17.06) | 17(10.00) |  |
|  | 5 (Rich) | 1,279(18.41) | | 19(8.48) |  | 33(19.41) | 15(8.82) |  |
| Household size | 1 | 1,052(15.15) | | 69(30.8) | 40.58  (0.000) | 38(22.35) | 33(19.41) | 16.14  (0.001) |
|  | 2 | 3,114(44.83) | | 79(35.27) |  | 21(12.35) | 51(30.0) |  |
|  | 3 | 1,305(18.79) | | 37(16.52) |  | 85(50.0) | 64(37.65) |  |
|  | $\geq$4 | 1,475(21.24) | | 39(17.41) |  | 26(15.29) | 22(12.94) |  |
| Health insurance status | National health insurance | 6,528(93.98) | | 185(82.59) | 48.38  (0.000) | 164(96.47) | 144(84.7) | 13.79  (0.000) |
|  | Medical aid | 418(6.02) | | 39(17.41) |  | 6(3.53) | 26(15.3) |  |
| Types of house | Single family house | 2,935(42.25) | | 90(40.18) | 12.34  (0.015) | 70(41.18) | 77(45.29) | 3.126  (0.537) |
|  | Multi-family house | 802(11.55) | | 38(16.96) |  | 24(14.12) | 28(16.47) |  |
|  | Row-house | 228(3.28) | | 7(3.13) |  | 5(2.94) | 4(2.35) |  |
|  | Rantal apartment | 2,786(40.11) | | 77(34.38) |  | 68(40.0) | 55(32.35) |  |
|  | Studio | 195(2.81) | | 12(5.36) |  | 3(1.76) | 6(3.53) |  |
| Home-ownership | Home owners | 5,188(74.69) | | 125(55.8) | 53.56  (0.000) | 126(74.12) | 99(58.24) | 14.15  (0.003) |
|  | Rental for year | 472(6.8) | | 22(9.82) |  | 14(8.24) | 17(10.0) |  |
|  | Monthly rental | 659(9.49) | | 51(22.77) |  | 12(7.06) | 34(20.0) |  |
|  | Provided housing | 627(9.03) | | 26(11.61) |  | 18(10.59) | 20(11.76) |  |
| Residential location | Ground | 6,868(98.88) | | 219(97.77) | 2.333  (0.127) | 168(98.82) | 165(97.0) | 1.312  (0.252) |
|  | Basement/Rooftop | 78(1.12) | | 5(2.23) |  | 2(1.18) | 5(3.0) |  |
| Region | Capital Seoul | 766(11.03) | | 32(14.29) | 3.291  (0.349) | 19(11.18) | 25(14.71) | 1.796  (0.616) |
|  | Gyeonggi-do | 1,190(17.13) | | 42(18.75) |  | 31(18.24) | 26(15.29) |  |
|  | metropolitan city | 2,050(29.51) | | 59(26.34) |  | 53(31.18) | 47(27.65) |  |
|  | Province | 2,940(42.33) | | 91(40.63) |  | 67(39.41) | 72(42.35) |  |
| Subjective health status | Good | 1,983(28.55) | | 23(10.27) | 161.21  (0.000) | 55(32.35) | 18(10.59) | 53.64  (0.000) |
|  | Normal | 3,432(49.41) | | 71(31.7) |  | 80(47.06) | 54(31.76) |  |
|  | Bad | 1,531(22.04) | | 130(58.04) |  | 35(20.59) | 98(57.65) |  |
| Health shock | No | 6,258(90.1) | | 178(79.46) | 26.68  (0.000) | 148(87.06) | 136(80.0) | 3.078  (0.079) |
|  | Yes | 688(9.9) | | 46(20.54) |  | 22(12.94) | 34(20.0) |  |
| Exercise ability | Good | 5,586(80.42) | | 108(48.21) | 143.11  (0.000) | 137(80.59) | 77(45.29) | 45.39  (0.000) |
|  | Mild | 1,323(19.05) | | 110(49.11) |  | 32(18.82) | 90(52.94) |  |
|  | Severe | 37(0.53) | | 6(2.68) |  | 1(0.59) | 3(1.76) |  |
| Self-management | Good | 6,422(92.46) | | 168(75.0) | 122.19  (0.000) | 161(94.71) | 129(75.9) | 24.03  (0.000) |
|  | Mild | 478(6.88) | | 43(19.2) |  | 7(4.12) | 33(19.4) |  |
|  | Severe | 46(0.66) | | 13(5.8) |  | 2(1.18) | 8(4.7) |  |
| Ability of Daily living | Good | 5,968(85.92) | | 133(59.38) | 176.3  (0.000) | 150(88.24) | 97(57.06) | 42.34  (0.000) |
|  | Mild | 927(13.35) | | 75(33.48) |  | 19(11.18) | 63(37.06) |  |
|  | Severe | 51(0.73) | | 16(7.14) |  | 1(0.59) | 10(5.88) |  |
| Pain | Good | 4,050(58.31) | | 59(26.34) | 131.29  (0.000) | 101(59.41) | 47(27.66) | 38.72  (0.000) |
|  | Mild | 2,761(39.75) | | 143(63.84) |  | 67(39.41) | 109(64.1) |  |
|  | Severe | 135(1.94) | | 22(9.82) |  | 2(1.18) | 14(8.24) |  |
| Anxiety or depress | Good | 6,017(86.63) | | 63(28.13) | 634.74  (0.000) | 154(90.59) | 45(26.46) | 144.2  (0.000) |
|  | Mild | 915(13.17) | | 151(67.41) |  | 15(9.4) | 120(70.6) |  |
|  | Severe | 14(0.2) | | 10(4.46) |  | 1(0.01) | 5(2.94) |  |
| Charlson Comorbidity Index (CCI) | 0 | 4,140(59.6) | | 115(51.34) | 6.405  (0.041) | 103(60.59) | 85(50.0) | 4.829  (0.089) |
|  | 1 | 1,685(24.26) | | 63(28.13) |  | 34(20.0) | 50(29.41) |  |
|  | $\geq$2 | 1,121(16.14) | | 46(20.54) |  | 33(19.41) | 35(20.59) |  |
| Disabled | No | 6,208(89.38) | | 181(80.8) | 16.42  (0.000) | 153(90.0) | 136(80.0) | 6.666  (0.01) |
|  | Yes | 738(10.62) | | 43(19.2) |  | 17(10.0) | 34(20.0) |  |
| Vision problem | Good | 3,932(56.61) | | 65(29.02) | 72.79  (0.000) | 95(55.88) | 44(25.9) | 31.68  (0.000) |
|  | Mild | 2,704(38.93) | | 135(60.27) |  | 63(37.06) | 107(62.9) |  |
|  | Severe | 310(4.46) | | 24(10.71) |  | 12(7.06) | 19(11.2) |  |
| Hearing problem | Good | 5,603(80.67) | | 132(58.93) | 65.55  (0.000) | 147(86.47) | 100(58.82) | 32.97  (0.000) |
|  | Mild | 1,058(15.23) | | 76(33.93) |  | 17(10.0) | 56(32.94) |  |
|  | Severe | 285(4.1) | | 16(7.14) |  | 6(3.53) | 14(8.24) |  |
| Oral problem | Good | 2,914(41.95) | | 34(15.18) | 99.94  (0.000) | 78(45.88) | 27(15.88) | 49.55  (0.000) |
|  | Mild | 2,066(29.74) | | 62(27.68) |  | 54(31.76) | 48(28.24) |  |
|  | Severe | 1,966(28.3) | | 128(57.14) |  | 38(22.35) | 95(55.88) |  |
| Memory problem | No | 6,610(95.16) | | 195(87.05) | 29.53  (0.000) | 161(94.71) | 143(84.12) | 10.06  (0.002) |
|  | Yes | 336(4.84) | | 29(12.95) |  | 9(5.29) | 27(15.88) |  |
| Bedridden | No | 6,721(96.76) | | 194(86.61) | 65.22  (0.000) | 163(95.88) | 149(87.65) | 7.628  (0.006) |
|  | Yes | 225(3.24) | | 30(13.39) |  | 7(4.12) | 21(12.35) |  |
| Depress | No | 6,615(95.23) | | 99(44.2) | 949.17  (0.000) | 164(96.47) | 77(45.29) | 107.8  (0.000) |
|  | Yes | 331(4.77) | | 125(55.8) |  | 6(3.53) | 93(54.71) |  |
| Psychiatric drug | No | 6,378(91.82) | | 142(63.39) | 212.75  (0.000) | 158(92.94) | 108(63.53) | 43.18  (0.000) |
|  | Yes | 568(8.18) | | 82(36.61) |  | 12(7.06) | 62(36.47) |  |
| Physical mental stress | No | 3,788(54.53) | | 19(8.48) | 483.44  (0.000) | 96(56.47) | 17(10.0) | 111.2  (0.000) |
|  | Sometimes | 2,823(40.64) | | 122(54.46) |  | 71(41.76) | 91(53.53) |  |
|  | Often | 335(4.82) | | 83(37.05) |  | 3(1.76) | 62(36.47) |  |
| Frustrating event | No | 5,267(75.83) | | 51(22.77) | 318.89  (0.000) | 133(78.24) | 44(25.88) | 93.34  (0.000) |
|  | Yes | 1,679(24.17) | | 173(77.23) |  | 37(21.76) | 126(74.12) |  |
| Unmet basic needs | No | 4,910(70.69) | | 46(20.54) | 255.72  (0.000) | 121(71.18) | 38(22.35) | 81.38  (0.000) |
|  | Yes | 2,036(29.31) | | 178(79.46) |  | 49(28.82) | 132(77.65) |  |
| Worrying about future | No | 4,004(57.64) | | 28(12.5) | 397.2  (0.000) | 110(64.71) | 24(14.12) | 105.6  (0.000) |
|  | Sometimes | 2,703(38.91) | | 135(60.27) |  | 58(34.12) | 102(60.0) |  |
|  | Often | 239(3.44) | | 61(27.23) |  | 2(1.18) | 44(25.88) |  |
| Smoking | No | 6,001(86.4) | | 189(84.38) | 0.75  (0.386) | 150(88.24) | 142(83.53) | 1.552  (0.213) |
|  | Yes | 945(13.6) | | 35(15.63) |  | 20(11.76) | 28(16.47) |  |
| Drinking | No | 3,088(44.46) | | 122(54.46) | 8.788  (0.003) | 71(41.76) | 95(55.88) | 6.78  (0.009) |
|  | Yes | 3,858(55.54) | | 102(45.54) |  | 99(58.24) | 75(44.12) |  |
| Total | | 6,946(100.0) | | 224(100.0) |  | 170(50.0) | 170(50.0) |  |
